# Miscellanies

**Published:** 1828-04-01

**Authors:** 


### MISCELLANIES.

#### 1. Royal College of Physicians.

We have learnt, with considerable surprise, that meetings of the Fellows of the Royal College of Physicians have been held, for the purpose of deliberating on measures to be taken against the Editor of this Journal, on account of comments lately published on the constitution and laws of that body. The Censors of the Royal College, in particular, have considered themselves as accused of dereliction of duty, and of venality in the discharge of their functions, by the observations made in the article on medical education, in the 4th fasciculus of this Journal. The writer of that article was particularly cautious in avoiding every expression that might, in the smallest degree, bear upon the personal conduct, or character of any individual, or class of individuals, belonging to the College. He confined his observations entirely to the effects of the constitution and laws of the College, (as handed down by their forefathers) on the progress of medical science—and did not even hint that these laws were either made, or badly administered, by the present race of Fellows or Censors. We understand that the Censors have considered our observations as reflecting on *them*, in their function of examining physicians for the licence, and as imputing to *them* certain mercenary motives, in passing over the said examinations, without proper attention to the qualifications of the candidates. No such interpretation can be fairly put upon our remarks. We argued, indeed, as an abstract principle, that the receipt of a revenue from the disposal of licences, in any corporate body, was a bad principle—" a principle which must incessantly *prompt* to extend the amount of sale, without *any* (we ought to have said *adequate*) reference to the qualifications of the purchasers." This is the head and front of our offending. We never said that any Censor, of this or that year, was seduced from his duty by the temptation which the laws of his college threw in his way. No. We carefully abstained from any such insinuation. We insisted on the evil *tendency* of the law; but never accused any set of men of giving way to this tendency. We have no hesitation, indeed, in saying, that the very Censor who has moved the College to proceed against us, has shewn that he could act in direct opposition to this evil tendency of the principle in question. So far from giving way to self-interest—or, what is the same, the interest of his College—he rejected one of the most eminent physicians of this metropolis, and thus deprived his College, for a time, at least, of the emolument derivable from the licence. The still more recent rejection of Dr. John Mason Good, was another example how far individuals will resist the influence of imperfect laws. But are we not to censure a bad principle, because there are men virtuous enough to act on an opposite principle? Are the laws of the College more sacred than the laws of our country? Does any one find fault with Mr. Brougham, for condemning laws enacted by that very senate of which he is a member? If no man be allowed to canvass or criticise a law that has once been passed—then no law can ever be abrogated afterwards. The College of Physicians have often altered their bye-laws—and surely this must have been on a conviction that they were not good. Why, then, should the College wish to destroy the liberty of the press, by persecuting a licentiate who investigates the tendency of other laws, not yet altered or repealed? We have never advocated the cause of revolution—we have never joined in the cry—" down with the charter," of this or of any college. No. We wish to see the original charter preserved in all its purity and simplicity—and we would certainly wish to see the bye-laws, instituted from time to time, in accordance with the spirit of the age in which they are framed. It was sagaciously remarked, by no less a personage than Sancho Panza, that—" it is safer for one man to steal a horse, than for another man to look over a hedge." So we have found it. Any word that drops from us, is taken up and canvassed with the most rigid scrutiny. But other writers may say what they please, with impunity. If we had put on our pages such a passage as the following—and that, too, from the pen of a most ardent advocate of the College,* the beadle would have been at our door in less than 24 hours!

" From the moment that the Corporation of Physicians submitted to this influence, (a recent influence which we shall not name) they lost the high ground on which their advocates had endeavoured to place them;—*they ceased to be the in-*

* Report says Mr. Fonblanc.

*flexible guardians of the principles transmitted to them by their predecessors. They had not adopted the reasonable rule, of relaxing the rigour of their bye-laws, in favour of* SUPERIOR TALENT; *but they sacrificed their independence to a petty intrigue.*"*

We ask the amiable and talented President of the College—we ask the Censors—we ask the Fellows, whether we have ever said any thing so severe as this?—The Editor of this Journal has only to observe, in conclusion, that if the President of the College will place twelve unbiassed and impartial physicians in the jury box, he will plead to the indictments which are about to be brought against him. But if he is cited before a tribunal, each individual of which unites in his own person the triple character of prosecutor, judge, and juror, he will be as mute as the marble busts of Harvey, Sydenham, and other of his departed brethren, ranged round the room where this scene is to take place. If the Editor has published, or suffered to be published, false facts or illegitimate conclusions, the press and the laws of the country are open to the College for redress. But, if the conditions of his licence preclude him from all freedom of thought and expression that may not be pleasing to the rulers of the said College, he will tender his license to the President, and fall *back* (if it be really a *retrogradation*) to that station in medical society, in which the greater and the best part of his life has been spent. To him, it matters not whether Dr. or Mr. be prefixed to his name. It is not the letter M, or the letter D— or the combination of these two letters, M.D., that can materially affect the residue of a life passed in the ardent (however unsuccessful) pursuit of medical knowledge. If the College, therefore, think that they shall enhance their authority or dignity by erasing his name from their list, he begs that they will not permit any private or personal feeling to interfere, for one moment, with the policy of the measure.

* The Jurist, No. III. Jan. 1828. In quoting this passage, we are far from agreeing with the writer of it, in his general principles. But we adduce the passage, to shew that even the College advocates can see defects in College administration.

### 2. DR. KELLIE ON TUBERCLES, AND THEIR EFFECTS ON DIFFERENT STRUCTURES.

(Dr. MONRO's Work on the Brain, vol. I.)

Dr. Kellie observes, that common tubercles may be formed—attain a considerable size—and long exist in any of the various structures of the body, without producing much trouble or sensible disturbance of function. But, when they become excited, inflamed, or suppurated, then they begin their work of disorganization in the contiguous parts, and produce trains of symptoms—more characteristic, however, of the injured function of the matrix of the tubercles, than indicative of the existence of the parasitic formations themselves.

"I have found tubercles in the serous membranes, and imbedded in the substance of the liver, of the spleen, of the kidney, of the uterus, of the lungs, of the brain, and of the cerebellum, in subjects in which no symptoms had indicated their existence during life. In young people, in children especially, from five to ten or twelve years of age, it is not uncommon to find tubercles of various sizes, and in various states of progression, co-existing in all or most of the organs in the same subject, who had enjoyed perhaps good, or very tolerable health, till within a very few days of his death. I found them in the brain, in the lungs, and in the peritoneum of one boy of seven years of age; in the brain, in the medulla oblongata, in the liver, in the spleen, and in the lungs of another boy at the age of nine; and yet these boys were active and well, and continued at school till within fourteen days of their death. Both died of Hydrocephalus acutus, passing, in the course of fourteen days, through all the stages of that disease, with nosographic regularity. In the latter boy, there occurred, indeed, during the last two days of his existence, a paralysis of the right side, and a spastic rigidity of the left arm, which enabled me to conjecture the probable existence, not of tubercles, but of inflammation and softening of the substance of the brain, as well as of the distension of the ventricles by serous effusion. Accordingly, besides the dropsy of the ventricles, I found two tubercles, each of the size of a garden-pea, hanging pendulous from the tentorium, two others of the same size in the medullary substance of the right hemisphere, and one oblong tubercle, of the size of an almond, suppurated in its centre, and imbedded in the pons varolii."

This fine boy, till attacked with headache, fever, &c. about a fortnight before his death, was apparently free from disease—was active, diligent, and clever at his school! The following case is interesting.

"On the morning of Thursday the 2d of August, about half-past eight o'clock, I was requested to visit, as speedily as possible, a boy of the name of Bell, residing in Poplar Lane, who had a little before been suddenly attacked with violent convulsions. When I arrived, the convulsive struggle was over, but there was still an occasional subsultus of the muscles of the face and of the arms. He was insensible and comatose; the pulse was very rapid, and the heart was striking against the side with remarkable violence. My inquiries were answered to the following effect: That his age was six years; that he had been upon the whole healthy, never had a convulsive fit before, was not liable to headaches, though he was to coughs ever since he had had measles; that he was in perfect health, in so far as could be discovered on Tuesday; that he had taken his food that day, and had amused and occupied himself as usual, going to bed at the usual time without complaint; that, on Wednesday morning, when dressing, he complained for the first time of pain of the loins, and of that only; that, continuing to do so, and seeming listless and unwell, his aunt had, towards noon, given him a dose of Epsom salts, which operated four or five times, but without relieving him; that, on the contrary, though he kept out of bed all day, he complained more and more of the pain in the back and loins till night; that, towards eleven, he fell asleep, but frequently awoke and complained of this pain, and at length, towards three this morning, he seemed to suffer so much, that Mrs. Nichol, a midwife, residing in the same quarter, and an intimate of the family, was called to assist him; that she administered an enema, and observing what she called a great working about the breast, she applied two leeches to the sternum; that the excruciating pain of the back continued unabated; that he complained incessantly, and was extremely restless till eight o'clock this morning, when the convulsive attack supervened.

"Six ounces of blood were instantly taken from the arm; another enema was then administered, and a purgative powder was directed to be given in a spoonful of gruel, so soon as the patient recovered the power of swallowing.

"But he never recovered his sensibility. The spasmodic twitchings of the muscles of the face and arms continued to recur at short intervals; and at half-past noon there came on another general convulsion, which continued, with little abatement, till one o'clock, when he died.

"Next morning the body was examined. The general appearance of the corpse was that of a well-grown boy of his age. The medulla spinalis, to which my attention was first directed, on account of the excruciating pain complained of in the back and loins, very carefully examined in its whole length, exhibited no trace of disease in its substance, its membranes, or its vascular system. The spinal nerves were equally sound.

"In the cerebrum I found three tubercles, and in the cerebellum one. Of those discovered in the brain, one the size of a small chesnut was found adherent to the arachnoid of the dura mater, and buried in the substance of the convexity of the posterior lobe of the left hemisphere, from which it was withdrawn along with the dura mater, on raising that membrane in the usual way from the cerebrum.

"When the softened cerebral substance torn from the brain by the removal of this tubercle was washed from it, the tubercle itself, of the size and shape nearly of a chesnut, appeared somewhat nodulated on its surface: it was very firm, of a greyish colour, and invested by a membranous cyst, having evident vascular and membranous connections with the arachnoid, and with the brain. Other two tubercles were found, one in each hemisphere of the brain, imbedded in the medullary walls of the lateral ventricles in corresponding situations, a little above and between the corpus striatum and thalamus of either side."

Dr. Kellie has not met with any other case, in which a fatal crisis has so suddenly occurred at so early an age. In young people, the more common termination of such cases is by consequent arachnitis and hydrocephalus—in adults, by inflammation and softening of the cerebral substance.

---

### 3. POPLITEAL ANEURISM—SECONDARY HÆMORRHAGE.*

What a happy thing it is for the critical tribe, that the surgical catalogue of ills, contains two such diseases as hernia and aneurism! They are positively a Journalist's stock in trade, and were they, by some dire mischance, but once obliterated from the nosologic chart, he might lay aside his gall and grey-goose quill for ever, and his occupation, like Othello's, would be gone! We have lately touched rather fully on the subject of aneurism, but it is so wide and debateable a ground, the questions, in many instances, so obscure, and the disputes upon them so hot, that—

> To count them all, requires a thousand tongues,
> A throat of *brass*, and adamantine lungs.

The subject of the present case, a coachman, æt. 40, was admitted into the Middlesex Hospital, on the 22d of October, under the care of Mr. Bell, labouring under popliteal aneurism. He had always enjoyed good health until three months previously, when he had an attack of gout, and about a month prior to admission, had first noticed pain and stiffness in the knee, which gradually increased. He was kept quiet in the hospital, until the 3d. November, when the swelling and tension of the limb having subsided, the operation of tying the femoral artery was had recourse to. The vessel was secured at the upper portion of the middle third of the thigh, without much difficulty, a small branch of the anterior crural nerve, which crossed it in front being cut away, and the ligature, which was single, "passed through the eye of a small needle, and brought through the integuments an inch distant from the incision, on the outside."† In the evening the bandage was a little stained with blood, but he went on very well until the 8th, when the house-surgeon perceived a slight pulsation in the tumour. This appeared for several days, more perceptible at one time than another, but never very distinct, and finally appears to have ceased. On the 17th, the ligature could be drawn out two inches, and on the 18th, it was found loose among the dressings. During the night, secondary hæmorrhage took place, and before the house-surgeon arrived, eight ounces of florid arterial blood were lost. The tourniquet was applied, and when Mr. Bell arrived, he prepared to tie the vessel, but on loosening the tourniquet no bleeding took place. He waited an hour more, and then left directions that compression should be employed if the hæmorrhage returned. Next day, the wound was cleaned, and a small clot found at its bottom, which was not interfered with, but the limb was rolled from the heel to the groin, compresses applied, and the part directed to be kept wet. On the 21st, every thing appeared favourable, when a flow of 'dark coloured blood' again took place from the wound, a roller was applied round the the thigh below the wound, which was enlarged upwards for about three quarters of an inch, the artery dissected for, and pulled out with a blunt hook: a ligature was passed around it, but not tied; the tourniquet loosened, but not a drop of blood appeared. After waiting some time, without any hæmorrhage, the ligature was secured, the wound dressed, and the limb rolled. On the 3d December, 14 days after the application of the ligature, it separated, the wound discharging a good deal. From this to the 15th January, he went on pretty well, the wound, however, being extremely indolent, and suppurating rather freely, but on the latter day, a hæmorrhage of what appeared to be venous blood took place. It soon ceased, and since that period has not returned, although the wound still remains fistulous.

The reader cannot fail to be struck by the similarity between this case, and the recent one of Mr. Brodie's, about which so much has been said. It is very difficult, perhaps impossible, to assign any satisfactory reason for the hæmorrhage in either case, but no doubt if the ligature had, by some chance or other, been applied in this instance, within an inch or so of the profunda, certain *impartial* gentlemen would have "ejected" their five or six pages of abuse on the occasion. Mr. Bell,

---

* Med. Gazette, No. 13.

† The object of this, we imagine, is—not to disturb the adhesion of the wound by the ligature lying in it.—*Ed.*

in a clinical lecture, delivered partly on this and partly on some other cases, has touched upon the question of hæmorrhage generally, and we shall take the liberty of noticing a few of his remarks.

Mr. Bell commences his lecture by some observations on the value of clinical instruction, in which we entirely agree, and only wish that it was more general than it appears to be. Mr. B. then alludes to the circumstances, both of them connected with death from uncontrolled hæmorrhage, which first drew the attention of the celebrated Guattani, and that modern "Tamerlane of surgery," as Dr. Barry very appropriately terms him, Sir Astley Cooper, to the study of their profession. These are pleasant topics, but alas! the *dulce* is not for critics, and we must pass them over, as we must a well told *hæmorrhagic* adventure of Mr. Charles Bell's own, occurring at—

> that dread hour,

> When church-yards yawn, and graves send forth their dead.

Mr. Bell asks himself, "how is a bleeding artery stopped?" and then proceeds to answer in the following manner. The blood is retained fluid "*solely* through the influence of the living vessel which contains it." This nescio quid, or living influence, according to Mr. B. resides in the inner coat, "for the moment the blood escapes from it, coagulation commences." This position is dwelt on again and again, and is illustrated by the well-known fact, that if an artery be merely cut or punctured, it will continue to pour out its blood ad deliquium, but if violently torn or contused, will frequently not bleed a drop. In the first instance, that of the clean cut, "the artery is opened without the disturbance of that principle of life, which preserves the blood liquid, and therefore the blood continues to flow," whilst in the latter case, we suppose the principle of life *is* disturbed, and therefore the blood does *not* continue to flow. In short, Mr. Bell conceives that the most important agent in the arresting of hæmorrhage, "is the influence of life which resides in the coats of the vessels and in the blood," and he laments, that this "essential" principle has been overlooked, and neglected by his contemporaries.

Now we are not among those who seem to consider the human body as a mere druggist's shop, a decoction going on in this organ, and a fermentation in that, but we do think that it is somewhat late in the day to revive the doctrine of the life in the blood. Upon the consideration of this theory we shall not enter, because, we believe, the opinions of medical men are pretty well decided on the subject; but we will just examine the power of this influence of life which resides in the inner coat of the blood-vessels. There cannot be a doubt that, in the greater number of aneurisms, the inner coat of the artery is ruptured, and this is a support, as far as it goes, to Mr. Bell's position. In many cases, however, the inner coat most certainly is *not* ruptured, and yet coagulation has taken place within the *dilated* vessel. Again, suppose the carotid artery dilated at its origin, and suppose a surgeon is called in, who resolves to immortalise himself by the operation of tying the vessel ultra tumorem. Well; the ligature is applied, the surgeon immortalised accordingly, and the patient dies, at the expiration of a certain space of time, of secondary hæmorrhage, from the upper portion of the artery. Upon dissection, the *dilated* portion of the vessel is found filled with coagulum, although the coats, inner and outer, are perfectly entire. We will take another instance; Mr. Bell, not liking the look of a ligature *ultra* tumorem, performs the operation for aneurism in the common way, or ties the artery on the face of a stump. This patient also dies, and the artery tied is found filled with coagulum, from the spot at which the ligature was applied, up to the origin of some large branch, a distance, we will say, of an inch and a half. These are not hypothetical cases, but in all of them is found a coagulum, and often a very considerable one too, where the inner coat of the artery was *perfectly entire*. Perhaps it may be said, "oh! but when the ligature is applied, an injury is done to the vessel, and the principle of life in its inner coat gets a shock, which disturbs the influence it naturally exercises on the blood." If this were the case, we should be glad to know why coagulation should stop at the origin of a large arterial branch, as when the femoral artery is tied immediately below the giving off of the epigastric, in which case there is little or no coagulum at all. But we meet with a still more unexceptionable instance of coagulation taking place within an artery whose inner coat is sound, namely, in what is called the spontaneous cure of aneurism. In this case, the coagulum forms not only in the aneurismal sac, but in the vessel leading to and from it, frequently to a considerable distance. From these facts it is evident that this living influence residing in the inner coat of the blood-vessels is purely imaginary, and that whenever the blood is placed out of the *current* of the circulation, be it in an aneurismal sac, a dilated artery, or merely a tied vessel, it coagulates, or, if you will, it dies. We are willing to allow that coagulation takes place more speedily and effectually out of a vessel than in it; but this is a very different thing indeed from that mysterious agency of a vis vitæ in the blood or blood-vessel, which it is so easy to talk about, and so hard to understand.

We have dwelt so fully on this point, that our limits will scarcely allow us to allude to the other preventives of hæmorrhage, namely, the retraction of the artery—the contraction of its orifice—and the formation of a coagulum within it. With regard to the first, it is exceedingly important, as every body knows, in its effects, and when prevented, as in the nutrient canal of a bone, &c. often occasions serious consequences. Mr. B. observes that he has seen, in compound fracture of the leg, the nutritia tibiæ divided, and bleeding so profusely, as to induce the consultants to believe that the posterior tibial artery was wounded. The contraction of the mouth of the vessel Mr. Bell makes to depend exclusively on the action of the muscular fibres, but we doubt whether the elasticity of the coats does not also come into play. Upon the coagulum, which forms at the mouth of a divided vessel, stretching within it and around it, and forming a kind of cap upon its extremity, Mr. Bell offers nothing new. He recommends the surgeon not to trust to it in amputation of the breast, &c. but carefully to tie each vessel, and encourage the bleeding at the time, by warm sponges and the administration of a little wine, so as not to expose the patient to all the alarm and suffering attendant on secondary hæmorrhage. In this sentiment, we believe, most surgeons of the present day are agreed.

### 4. Chloride of Lime.

Mr. Davidson, of Glasgow, has published a few observations on this corrector of putrid effluvia in the first number of Mr. Mackenzie's Journal.

The following extract contains the particulars of this communication.

"It is an opinion entertained by many medical men, that the fœtor of intestinal gases depends upon the presence of sulphuretted hydrogen. Now, though it had not been proven by analysis, that sulphuretted hydrogen is seldom found a constituent of these gases, yet analogy would lead us to conclude that their fœtor depended solely upon the presence of putrid effluvia. For, if a portion of excrementitious matter be mixed with a solution of chloride of lime, the fœtor is instantly destroyed, in the same way as it would be, if the solution were applied to a sphacelating ulcer, or mixed with putrid oil. That putrid effluvia are totally different from contagious miasmata, is very evident; because the first can, with great facility, be detected by our senses, and exist in bodies which have no infectious properties; whereas the existence of the latter can only be inferred from inductive reasoning. And it is a begging of the question to maintain, that because chloride of lime destroys putrid effluvia, it must therefore necessarily destroy contagious effluvia, because both exist at the same time in the same animal body. It may be true that the chloride of lime can destroy contagious effluvia; but till it be demonstrated by rigid experiments and observations, we have no right to draw conclusions from such vague and unphilosophical analogies.

"There is no substance which destroys putrid effluvia so thoroughly as chlorine, or its compounds. Chloride of lime was first used in France, for this purpose, which certainly forms not one of the least important applications of this extraordinary compound.

"The following account is an abstract of a few cases in which the chloride of lime has been used:—J. S. has been affected with cough and expectoration for about six years, and is considerably emaciated. On the 25th October, 1826, he was affected with sloughy ulcerations in his gums, which emitted a very fetid odour. A gargle was prescribed, in the proportion of two grains of chloride of lime to an ounce of water. This, used occasionally, kept the fetor in check, and the ulcerations cicatrized in about eight days. 2d. Mrs. C. in November, 1826, used mercury for a hepatic and dropsical affection, to the extent of copious salivation. A gargle, in the proportion of two grains of chloride of lime to an ounce of water, was used; which produced considerable smarting. It was afterwards reduced in strength, and a portion of refined sugar was added, which removed in a great measure its harsh taste. It was continued for a fortnight, and when used frequently, always had the effect of moderating the fetor. The same treatment has been adopted uniformly in all other similar cases, and with similar effects. 3d. Mr. M. was affected in December, 1826, with diarrhœa; fæces dark and very fetid. One teaspoonful of chloride of lime was ordered to be dissolved in about sixteen ounces of cold water; and the fæces to be discharged into the solution. He used it in this way for ten days, and affirmed, that in all cases it had the desired effect of destroying the fetor. This plan has been adopted in a variety of cases, during the late dysenteric epidemic, and in several cases of fever. 4th. A boy about twelve years of age has expectorated about four ounces of pus every day for three or four years. It seems to be discharged from a cavity in the right side of the chest; for the sound emitted on percussion is very dull in that situation. He expectorates the whole of the pus in a few minutes, and it occasions a very disagreeable fetor in the apartment. But by discharging it into a strong solution of chloride of lime, and afterwards gargling his mouth with a similar solution, much of the disagreeable fetor is destroyed. 5th. A blacksmith in Nov. 1826, received an injury on one of the joints of a finger, which produced extensive and fetid suppuration of the joint. The fetor was completely destroyed by moistening the dressings, (before they were removed,) with a solution of chloride of lime, in the proportion of two grains of the salt to one ounce of water. 6th. Mrs. V. was delivered in February last of her first child. The labour was very tedious, and there was high excitement during the latter stages. On the third day after delivery, she was affected with fixed pain in the uterine region, and very fetid lochial discharge. A solution of about two grains of chloride of lime to an ounce of water, was ordered to be injected into the vagina, two or three times a day. This treatment removed the disagreeable odour; and she completely recovered in about ten days. 7th. Mr. M. whose breath is naturally fetid, was directed to gargle his mouth and brush his teeth with a solution of chloride of lime, in the proportion of four grains of the salt to an ounce of water, with the addition of a small portion of refined sugar. It had the effect of removing the fetor of the sordes which accumulated about his teeth. 8th. J. L. died of diseased liver and dropsical effusion into the abdomen. On laying open the abdominal cavity, a very fetid odour made its escape; and adhered very tenaciously to the hands. After the dissection was finished, they were thoroughly moistened with a solution of chloride of lime, in the proportion of about six grains of the salt to an ounce of water, and afterwards washed with soap and water. To medical men, this is an important application; for after dissections, the fetor often adheres to the hands, most tenaciously, for many hours. A solution of a similar strength was poured upon the body, and the clothes in which it was wrapped were also moistened."

Dr. D. strongly recommends the use of this substance, both in public and private practice.

"In ptyalism," says he, "it is frequently of great consequence to many a young man, to remove the fetor of his breath, and to prevent the consequent discovery of his disease; and even where there is no reason for concealing the use of mercury, how agreeable to the feelings of the attendants would be the neutralization of such offensive putridity!"

---

### 5. Fever.

Three evenings of the Westminster Medical Society have been taken up in the discussion of the nature, seat, and treatment of fever. The subject was brought forward by Dr. Barry, on the basis of Dr. Clanny's experiments on the blood of typhous patients, alluded to in the 4th

Fasciculus of this Journal, and the humoral pathology was ably maintained by the former gentleman. We are quite unable to enter into the details of the arguments brought forward by the Humoralists and Solidists, on this occasion. All were agreed on one fact—the vitiation of the fluids in the course of the fever; but the grand point of contention turned on the question of priority, as to the nervous or vascular systems being the primary seat of fever—or, in other words, did the remote cause, say contagion or malaria, make its first impression on the nerves or on the blood-vessels? Dr. Barry, of course, argued for the latter; while Dr. Copland and Dr. Thomson contended for the neurological pathology of fever. In the second night's debate, Dr. Copland took an opportunity of fully developing his doctrine of fever, which supposes the morbific cause to make its first impression on the system of ganglionic nerves —especially on those distributed to the air passages in the lungs. From this source, he conceives, the morbid impression is transmitted to the cerebro-spinal system of nerves, and consequently that all the functions dependent on both systems become ultimately deranged—and the fluids contaminated. Dr. Thomson maintained, that the first impression was made on the nervous system—and that all the derangements of function and vitiation of fluids which followed, were *effects* of this morbid state of the nerves. Dr. Johnson argued, that an accurate observation of the phenomena which presented themselves to the senses, after the application of the cause of fever, whether a contagion, a specific poison, as that of small-pox, plague, &c. or a malaria, would not enable any unbiassed man to determine, that the nervous system was more particularly or more early implicated than the vascular apparatus. It was true, the phenomena of sensation came more clearly under our view at the beginning of fever, than any changes in the fluids could possibly do. We could hear a man complain of lassitude or pain in his limbs, but we could not see any poison that might have been floating in his veins, and acting on the nervous system for days previously. The period of incubation, or that period of apparent health which intervened between the application of a poison, say small-pox contagion, and its open manifestation, indicated that some contamination of the fluids was going on secretly all that time.

Mr. North, Dr. Ley, and Mr. Lambert declared their opinions, that the subject of discussion was totally useless, and could never bear, in the remotest degree, on our practice at the bed-side. This damper on the "march of intellect" was somewhat indignantly met by Dr. Barry and Dr. Thomson, who, though opponents in the controversy, now joined heartily in defending the propriety—the utility of discussions on the nature, seats, and causation of diseases, even although such discussions might not lead to a complete elucidation of any of the points discussed. In this sentiment the majority of the Society seemed to agree.

In the course of this discussion some therapeutical observations came out, which are not undeserving of attention. Dr. Copland advocated the propriety of emetics in the incipient stage of fever, as a remedy by which he had, in numerous instances, arrested at once the progress of the disease. On the other hand, he had seen oil of turpentine save the lives of great numbers, in the last stage of exhaustion, when no other remedy offered the slightest chance of success. Dr. Barry adverted to the very opposite plans pursued by the Portuguese physicians and the disciples of Broussais. In the Portuguese hospitals, there is an emetic room, into which the fever-patient walks, as a matter of course, when he first arrives, and there undergoes the necessary discipline. After the emetic, he has chicken-broth, and saline draughts alternately, through the whole course of the disease. Bleeding or any strong medicine is rarely thought of—and the general result is as favourable as under the most vigorous depletive measures of British practitioners. On the other hand, he has followed M. Broussais for weeks and months —he has seen his patients starved or leeched to death, so that, on dissection, any man might have read the smallest print through the coats of their intestines! Broussais, of course, abhors the Portuguese emetic plan. Indeed he generally promises to cure all those who have not been poisoned by emetics and purgatives. Seeing, then, such diversities of opinion, and such empirical modes of treatment pursued, was it not (he said)

desirable to calmly investigate the seats and causes of fever, in order that the minds of youth and of practitioners might be emancipated from the fetters of empiricism at least, if they could not arrive at the exact truth?

In the third night's discussion, the debate was principally carried on between Drs. Ley, Copland, and Barry. Therapeutics formed the prominent subject of investigation, and the efficacy of oil of turpentine in puerperal fever was warmly advocated by Dr. Copland, who seems to have had great success with this remedy in the latter stages of what he called *malignant* puerperal fever. He seldom gave more than half an ounce or an ounce in a draught—or double that by enema—and, in general, he did not give it more than twice in the same case. Fomentations of the same were also used. Dr. Ley seemed to view the remedy as one which relieved a particular and very distressing symptom of the disease—tympanitic distention of the abdomen. For such it was an admirable remedy; but he did not appear to consider the oil of turpentine as possessed of any specific powers against the dangerous disease in question. The utility of theory in fever was again agitated in the Society, and Dr. Barry, Dr. Copland, Dr. Ley, Dr. Johnson, and others, took active parts in the discussion.

---

### 6. Literary Justice.

Our readers will remember the charge made by Mr. Lawrence against Dr. Johnson, in our 4th Fasciculus, and Dr. Johnson's answer to that charge. The Lancet, with its usual justice, publishes the *charge*, but suppresses the *reply!* In place of the latter, the Lancet favours its readers with TEN COLUMNS of blackguard abuse of Dr. Johnson, including, at least, one whole column of downright falsehoods! If we gave the Lancet a large weekly fee for enhancing the character of this Journal and damning its own, no surer method of effecting both objects could be pursued than that which it has adopted. We earnestly entreat each and every of our readers to peruse the twelve columns (it always dedicates twelve columns to us) between page 448 and page 454, of the Lancet, No. 236, as the greatest compliment that this Journal has ever received since its first commencement. In what a predicament has the Lancet placed Mr. Lawrence! It publishes *his* statement—suppresses *ours*—and substitutes ten columns of abuse, lies, and garbled extracts! "There is such a thing as common sense;" and although there may be no justice in the Lancet, there is justice in the minds of professional men. For this last act we return unfeigned thanks to the Lancet. That journal has done Dr. Johnson more real service than all his friends put together. Perge pede quo cepisti.

---

### 7. Erysipelas.

Sir A. Carlisle has published some sensible remarks on this disease in the 14th number of the Medical Gazette, not, however, unmixed with some fanciful opinions. We find him an advocate for the union of physic and surgery—(a union which we have long advocated)—for the constitutional origin of erysipelas—for the humoral pathology of the disease (as "arising from vitiated blood")—and for its occasional epidemic character. "Assuredly (says he) the common source of erysipelas is improper diet, through which the blood is supplied with crude or noxious materials." Sir A. is fully convinced that excitement in the system, as from catching cold, only "gives activity to a previously existing morbific humour."

"Those violent eruptions, vulgarly, but not illogically, called surfeits, are the immediate consequences of one pernicious meal; and they generally display Erysipelas in its most malignant character; while moderate and habitual errors in diet mitigate the acuteness of the disease, and keep the patient in a state of continued liability to relapses, whenever exposed to any extraordinary vicissitude, and which thence assume either a mild or chronic type."

It must be allowed (as Sir A. justly remarks) that erysipelas is frequently *first* observed as a local affection, and apparently limited to the corion or true skin; "but the eruption is *always* preceded by some derangement in the alimentary functions, and, for the most part, by loss of appetite, nausea, and constipation, followed by the other signs of incipient fever." As far as idiopathic erysipelas is concerned, we believe Sir A's observations are correct; but there are cases of traumatic erysipelas, where we cannot always trace this preceding disorder of the digestive apparatus. A man, not perhaps in sound health, or of an irritable constitution, receives a wound or hurt, and a few days afterwards, erysipelatous inflammation spreads from the injured part. "Perhaps all the varieties of erysipelas, says the author, are to be ascribed to the several deviations in the acrimony, or in the quantity of the exuding morbid humour, and in some degree, to the constitutional habit of the patient." Sir A. having examined the serous effusion under the skin, in a great number of cases of erysipelas, "always found it to contain a free acid." The following, in a few words, is Sir A's pathology of the disease in question.

"My views of Erysipelas, thus matured, are, that it is a humoral and constitutional inflammatory disease, occasioned by alimentary crudities; and because certain vegetable acids, and acidifiable viands, are often the notorious antecedents of the disease. I believe, as before stated, that the dominant error in the morbid fluids is acid."

We shall subjoin our author's therapeia. He does not like venesection, and prefers cupping, in plethoric habits; but the following is the plan of treatment on which he seems to depend.

"When the attack has been sudden, and the stomach is known to be loaded with crude food, an emetic of ipecacuanha is adviseable, and it should be immediately followed by a cleansing cathartic, given in reiterated doses until the lower bowels are thoroughly emptied. If the patient be of a full habit, and the pulse ample, abstinence from food and drink should be recommended, because the functions of the stomach are especially deteriorated in Erysipelas, and diluents promote all the alimentary fermentations. Mercurial purgatives are objectionable, because they produce putrescent excretions; and also, because of the uncertainty as to their injurious constitutional effects on certain individuals. Saline cathartics seldom cleanse the bowels effectually, and they mischievously augment the fluidity of the aliment.

"The purgative which I ordinarily advise is composed of one drachm of powdered jalap, two scruples of sulphate of potash, and half a drachm of carbonate of soda, mixed with eight ounces of infusion of senna; of this two or three table-spoonsful should be taken every second or third hour, until copious and cleansing evacuations are procured. From a conviction that alimentary acidity is generally prevalent and requires to be subdued, I give from ten to sixteen grains of sub-carbonate of soda every four hours, in barley water. If the mouth is parched, soda water proves very refreshing. Where great debility is obvious, the carbonate of ammonia, in doses from five to eight grains, should be preferred to soda; and three or four grains of aromatic powder may be joined with each dose."

As might be expected from the doctrine maintained by Sir Anthony, the local treatment of erysipelas is a very subordinate consideration. Free incisions are recommended, where the subcutaneous tissue has been "invaded by the morbid secretion." When sphacelus has taken place, the chloride of lime is spoken of in terms of the highest applause. "This estimable compound arrests the putrefactive fermentation; by which not only the local condition of the mortified parts, but the general health and comfort of the patient, are essentially benefitted." Dietetic regimen is strongly insisted on by Sir Anthony. Although much inclined to the humoral pathology, we can hardly help suspecting that the acid theory of the author is more fanciful than sound We agree with him in the general principles of treatment, excepting his futile objections to mercurial purgatives; and also in the view which he takes of the constitutional origin of the disease.

---

### 8. Mr. Abernethy's Lectures.

These lectures are now published through two, if not more channels—and they are still delivered, annually, to the largest class of students that is to be seen in this metropolis. That the doctrines, opinions, and practices promulgated and inculcated through these lectures, must have a very extensive influence on the profession, especially on the junior orders, cannot be questioned; and if the matter of the lectures presents legitimate subjects of discussion, or even criticism, we think that no publication can better deserve an examination than that which is most universally read and most generally influential. We shall, therefore, take up the various diseases treated of in these lectures, promising that our comments shall be guided by no other feeling than a desire to establish truth.

### Physic and Surgery—Fever.

Mr. A. upholds the "unity and indivisibility" of physic and surgery—and then goes on to maintain, that the symptomatic fevers arising from local injuries are "exactly like those which Cullen has described under the heads of synocha, synochus, and typhus—and no physician can distinguish them from those which occur without any external injury." Again, he says—"I will venture to assert, that the fevers produced by local disease, are the very identical fevers which physicians meet with where there is no external injury." This doctrine we hold to be equally erroneous and dangerous. It presumes that the causes of fever make no difference in the character of the disease. This is a great error. Look at two men, one of whom receives a bayonet-wound in the chest—the other a malaria in Walcheren—or the contagion of typhus in the purlieus of Saffron Hill. Will the period of incubation, the invasion, the nervous and vascular phenomena, the duration, or the termination of the two fevers be *identical?* No! Is the fever resulting from the contagion of typhus, or the miasm of the fen, as much within the control of art, as the symptomatic fever arising from the wound in the lungs? Can we "put out" the typhus fever in two, three, or four days, by venesection, not measured by ounces, but by wash-hand-basins full? No, verily. If, then, the character, the course, and the treatment of the idiopathic and the traumatic fevers, are different, as we maintain them to be, how is Mr. Abernethy justified in the sweeping assertion, that they are identical? It is very true that, towards the fatal close of all fevers, whether symptomatic or idiopathic, the features approximate, and the character of the morbid phenomena becomes nearly, if not wholly, identical. But this is no fair reason for confounding them all together. The very description which Mr. A. gives, at page 7 of his first lecture, negatives its application to the great class of malarious and contagious fevers. There is no similitude between the symptoms of these last, and those resulting from a compound fracture, as delineated by our author.

Mr. Abernethy next proceeds to what is called the "*ratio symptomatum*" of sympathetic fever, which is summarily dispatched in the following manner. "Hang it, I need not trouble you with this; the symptoms explain themselves; they are the natural *consequences* of the excitement of the heart and arteries, *and the disturbed state of the digestive organs*." This is certainly a very easy way of cutting the Gordian knot! The whole *ratio symptomatum* in fever is explained by excitement of the heart and arteries—and disturbance of the digestive organs. Whether this disturbance is a cause, or an *effect*, of the local and constitutional affection, we leave our readers to judge. It would not be worth our while to enter into an examination of this point, in the present instance. The treatment is still more easily managed.

"Treatment! there is no treatment. The disease is the necessary and natural consequence of the injury: it must inevitably take place:—You may mitigate, but you cannot cure it." What! there is no treatment—yet "you may mitigate" the disease! Is this not the *treatment* in the great majority of diseases? Can we in any case, take the ratio medendi out of the hands of Nature, and cure the disease by the main force of art? The lecturer goes on to say, that we must bleed in symptomatic fever; but really his observations are calculated to destroy the manufacture of lancets, to starve the increasing population of cuppers, and to revive the trade of leech-catching!

"Do not take away his blood, which is his life, for you may find after a certain time, that he will stand in need of every degree of vital energy to recover from the injury. I have seen a patient bled, and bled, and two or three days after, the medical man has been glad to throw in the bark, and try every means, when it is too late, to give strength and vigour to the constitution of the patient."

Again, Mr. Abernethy says :—

"As the *fever subsides spontaneously on the third or fourth day*, when suppuration takes place, avoid bleeding the patient if possible. Give him diluent drinks and acids, such as lemonade, &c. for which he has a desire."*

Cleansing the bowels is properly insisted on, as a necessary measure. But, as the lecturer grounds his observations on a case of *compound fracture*, and that in a London subject, where he does not think a healthy subject is to be found, it is manifest that the didactic precepts, as far as regards fever, are extremely imperfect—not to say erroneous. London is not spread over the whole surface of the earth—and injuries are not confined to compound fractures of the legs of drunken draymen in the vicinity of Bartholomew's Hospital. What would have become of our soldiers and seamen in the late war, if the fever induced by the wounds of bayonets, bullets, and splinters, had been treated according to the *economical* principles inculcated in these lectures? If our medical officers had acted on these narrow principles of economy, in respect to blood-letting, we can only say that they would have verified the old adage—"penny wise and pound foolish." The lecturer remarks that purgation, by promoting the secretions, lessens the plenitude of the blood-vessels. "This is a fact which *those who have not studied anatomy and physiology cannot be so thoroughly acquainted with as those who have.*" The ancients were well acquainted with this fact, although very imperfectly versed in anatomy and physiology. But leaving this on one side, who are those against whom the above sarcasm is levelled? Against PHYSICIANS, of course! They can be supposed to know nothing of anatomy and physiology. Yet if we go into the medical societies of this or any other metropolis, we shall find that physicians are not behind the level of their surgical brethren, in anatomy, physiology, or pathology. No class of the profession should be singled out for animadversion. He who cultivates his art should be reverenced, be his designation what it may—he who does not keep himself on a level with the progress of science should be stigmatized, even if he were a teacher of the art. Medical knowledge is not restricted to names, grades, or ranks. It is a REPUBLIC in which TITLE will not *always* screen ignorance, nor HUMILITY *always* prevent the rise of merit.

* Is it not astonishing that a man, professing to be an accurate observer of nature, and a critic on the opinions of the most eminent physicians of the world, should maintain that a fever "*which subsides spontaneously on the third or fourth day*," is *identical* with that which physicians meet every day where there is no external injury—in other words, that this fever from a wound is the same as that from contagion or malaria!! Is this a doctrine to guide the rising generation of pupils, when they go into the country to practise their profession?

It is true that Mr. A. apparently recollecting himself, says—"if a man be shot through the body—if an internal and vital organ is injured and inflammation come on that organ," you must bleed repeatedly and decisively. "We do not, however, bleed here for fever, but for inflammation." Now by this passage, Mr. A. virtually gives up the identity of idiopathic and symptomatic fevers. According to this doctrine, we have no warranty for venesection, however high the symptoms of fever may run, unless we have evidence of inflammation of a vital organ! We are not to bleed for fever—but for inflammation. Mr A. ought to know that most violent fevers commence without inflammation—and if we did not bleed, in such cases, inflammation would assuredly supervene in some organ or structure. The doctrine is decidedly bad.

### 9. INFLUENCE OF THE STOMACH ON THE BRAIN.

In a former paper we gave some account of a dissertation by M. Bayle, on the influence of chronic inflammation of the stomach and bowels in the production of mental alienation. In a second memoir, he adduces further examples of this influence, and winds up with certain conclusions or reflexions, which we shall briefly notice.

1mo. In a few cases, chronic phlegmasia of the mucous membrane of the stomach and bowels, is capable of inducing mental alienation—and, in *many cases*, it is capable of keeping up the mental malady, and modifying its character.

2ndo. Most of those who become deranged at the close of chronic gastro-enteritis, have inherited a constitutional predisposition to the disease from their parents. Their mental faculties are generally weak—the brain irritable. The dyspeptic and nervous sufferings of people labouring under gastric and intestinal irritation, tend very much to mental hallucination.

3tio. The anatomical characters of the gastro-enteritis which accompanies or causes mental alienation, does not differ from that which is unaccompanied by any mental aberration.

4to. The manner in which the gastro-enteric affection acts on the head is two-fold. When the pain of the abdominal malady is great, it is sympathetically propagated to the brain, and there disturbs its functions, in the same way as it is propagated to the heart, and produces the phenomena of fever. Hence the symptoms of mania. But when the gastro-enteritis is chronic, and less intense, or the cerebral system of the patient less disposed to irritation, then the dyspeptic sufferings of the individual gradually dispose to hypochondriacism—and hence arise various kinds of monomania—and especially the dread of being poisoned, with obstinate refusal of food, &c. These two forms differ only in degree, and they often pass from one into the other.

5to. This dread of poison and obstinate refusal of food are regarded by M. Bayle as the most constant and essential symptoms of mental alienation accompanied by chronic inflammation of the mucous membrane of the digestive tube. The two symptoms above-mentioned are "the expressions of gastric and intestinal sufferings in mental alienation." They may exist, he observes, without this condition of the stomach or bowels, but he has never seen a case where they were present, and where the symptoms of chronic gastro-enteritis did not shew themselves during life, or the traces of it were found after death.

6to. It follows from these premises, if they are correct, that the treatment of insanity, where these phenomena obtain, must be specially directed to the gastro-intestinal affection—and does not differ from that which is necessary or proper where no cerebral disturbance is complicated with the original malady. This treatment hinges almost entirely on repeated leechings of the epigastrium—mucilaginous drinks—extremely abstemious regimen—exercise in the open air—counter-irritation.—Revue Medicale.

Fanciful as the above doctrine may appear to those who look no farther than the organ *apparently* most affected, for the source of a disease, we believe there is some foundation for it in fact. The mental miseries resulting from gastro-intestinal irritation are but very imperfectly known even to the most experienced physicians. Nothing, in short, but actual personal sufferings can teach the terrible but instructive lesson !

---

### 10. Irritability of the Air-cells—Asthma.

A perusal of Mr. Abernethy's lectures, as now published through more than one channel, is calculated to excite various emotions. There is a mixture of accurate observation, eccentric, not to say erroneous deduction, shrewd remarks, and humorous naiveté, which is altogether unparalleled in didactic instruction, and medical literature. It is our intention to give a running commentary on these lectures, which we hope to render somewhat interesting. The present subject is irritability of the mucous membrane of the air-cells, as one of the principal causes of that mysterious disease asthma.

Of the irritability of this membrane Mr. A. entertains no more doubt than of the irritability of the urethra—of which there can be no doubt.

"Ordinarily, respiration may be said to be a mechanical process ; we enlarge the capacity of the chest by the *intercostal muscles*, and the air is forced into the lungs : we diminish the chest, and the air is forced out of the lungs, just as if you were using a pair of bellows. Ordinarily, respiration is carried on merely as a mechanical process ; but, extraordinarily, do we not find manifestations that air cannot get into the lungs, though we do endeavour to enlarge the chest? You know you never could lift up the board of the bellows, if you were to stop up *the holes that admit the* air, and why? On account of the immense weight of the *atmosphere*. Just so, if you were to put a rope round a man's neck, and stop the air from entering into the trachea; it is not the strongest man that ever lived that could afterwards enlarge that man's chest; to do that, would be to lift up an immense load of air. A man having irritable lungs, may be sitting comfortably enough at the fire-side, but a little smoke comes into the room, and he can breathe no more: he gasps for breath, he cannot enlarge the chest, and he finds the utmost difficulty in respiring; but where is the difficulty? Where is the sensation of pain and contraction? Why, in the lungs themselves; the hindrance is there; I believe it is all irritability."*

Passing over the error that respiration is carried on by the intercostal muscles alone, we cannot but see the want of analogy between the bellows and the lungs in this case. If, indeed, Mr. A. had said that irritation of the mucous membrane of the glottis, might throw the surrounding muscles into a state of spasm or contraction, and thus prevent the ingress of air into the lungs, we could readily understand him; because it is in this way that irritation of the urethra causes temporary or spasmodic stricture of that canal. But, when he says that irritation of the mucous membrane lining the air-cells causes contraction of the said cells, and shuts out the air, we are at a loss for the *quo modo*—unless he can demonstrate muscular fibres in the parietes of these cells. We are inclined to think that the true explanation will be found in irritation of the membrane lining the passages leading to the lungs (and which, as we said, are surrounded by muscles) rather than in the air-cells themselves. Be this as it may, it is very difficult to account for the phenomena presented by what are termed asthmatic people. Pure air *generally* relieves the dyspnœa, where there is no organic disease—but not always. Asthmatic people of London go about the streets "with their shoulders hitched up to their ears, and using every auxiliary to enlarge their chests." Mr. A. instances the case of a neighbour whom it was painful to see walk about, while breathing an impure air, as he appeared to be in imminent danger of suffocation. Such was his state in Bedford-row, but by the time he had got to the top of Gray's Inn-lane, in his way to his country-house, for a mouthful of fresh air, "he breathed perfectly well." But there are many others who breathe better in an impure than in a pure air. Mr. A. knew a man, "whose lungs were so asthmatical that he could never lay down in his bed at night," and he was advised to go to the South of France. He consulted Mr. A. "I told him what I tell every body else, that the best thing he could do was to take care of the state of his stomach." In about three weeks, he called again on Mr. Abernethy, and reported that he had done according to his directions, and that he had breathed the air of London for the above-mentioned period of time, without the least difficulty of breathing. He went back to his native air, which was on the top of a high hill—and he was nearly suffocated the very first night he slept there. Another man lived in a room which was filled with sulphuric acid gas, and found that "it relieved his difficulty of breathing in an amazing degree."

"I say that is whimsical; but all this leads to convince me, that there is a state of irritability in the lungs, and which proceeds from the state of the stomach too."

It is almost needless to say, that this physiology and pathology of the mucous membrane of the bronchia and air-cells, is extremely imperfect, and far from what we would expect from such a man as Abernethy. When we find a paroxysm of spasmodic asthma, as it is called, lasting for one, two, or three days, and ending in a copious expectoration, we cannot but conclude that a turgescent condition—a congestion, if you will—of the vessels distributed over this extensive membrane, had previously existed—and that Nature had relieved the dyspnœa, by the discharge of a copious effusion or exudation from the said membrane, in the shape of a muco-purulent expec-

* Lectures, page 375.

toration. The doctrine of mere irritation then, is, like many other doctrines, too confined and exclusive. It is not founded on an extensive and liberal view of the phenomena presented by disease.

---

### 11. DIAGNOSIS OF HERNIA.

A very animated discussion on this point took place at the Westminster Medical Society, on the evening of the 15th, and really some observations fell from one or two gentlemen on the occasion, which, as the Americans have it, would be exceedingly important, *if true.*

The subject was very ably introduced by Mr. Cæsar Hawkins, who considered, seriatim, the symptoms diagnostic of hernia—the tumours which may be mistaken for it, or combined with it—the contents of the sac, where a hernia has been proved to exist—and, lastly, these having been ascertained, their pathological condition. From this it is evident, that the field was a very extensive one, and we are sorry that our limits will not permit us to do justice to Mr. Hawkins' observations. With regard to the symptoms of strangulation, Mr. H. thought that it was exceedingly difficult to distinguish between them and inflammation of the intestines, or ileus, in *acute* cases; but, in the chronic forms, the diagnosis is by no means so difficult. Varicocele, or varix of the veins of the round ligament, a very rare disease, of which we detailed a case in our last fasciculus, is not unfrequently mistaken for omental hernia; but, by placing the patient in the erect position, and making pressure at the ring, the descent of a hernia is prevented, whilst the varices, if they exist, become turgid. Enlargement of the femoral vein, too, beneath the crural arch may counterfeit a femoral hernia. The diagnosis, however, is similar to that in the former instance, viz. pressure on the groin, which causes the vein to swell, but prevents the descent of a herniary tumour. Hydrocele, encysted hydrocele of the cord, especially the latter, and an enlarged absorbent gland, which is situated just within the external ring, may be severally mistaken for inguinal hernia, whilst it is well known that surgeons, even of eminence, have cut down upon a bubo, imagining it to be a femoral hernia. After noticing various other sources of error to which the practitioner is exposed, Mr. Hawkins proceeded to point out the distinctions between intestinal and omental hernia, which he thought of importance, especially in the more chronic cases. In the former, when the intestine is closely girt, purges maybe useless, or worse; whereas, in epiplocele, they may prove of service, and, at any rate, we have more time afforded us, before having recourse to an operation. In the acute cases of intestinal hernia, Mr. Hawkins was of opinion that bleeding and the warm bath should precede the application of the taxis, as they tend to diminish the inflammatory engorgement of the tumour.

Dr. Somerville remarked that he had frequently seen patients die after the operation for hernia, performed apparently under favourable circumstances, when no organic lesion of any consequence could be detected after death. In such cases, the nervous system appeared to have received a shock, and the patients to have sunk under that state which has been designated by the name of "constitutional irritation." This was a most unfortunate phrase, for no sooner was it uttered than Mr. Lambert started on his legs, and expended we know not how much valuable argumentation, to prove that this "constitutional irritation" was a mere non-entity—an ignis fatuus in the Doctor's brain. Mr. Bennet observed that there is most certainly "a something," call it what you will, which supervenes even on trifling accidents or operations, and carries off the patient quite independent of any inflammatory process. Thus, in opening the abdomen of dogs, Mr. B. had seen them die in so short a period, that it was impossible for inflammation to have developed itself.

Dr. Duffin having related a case in which he had nearly mistaken an enlarged and suppurating gland, under very obscure circumstances, for an inguinal hernia, was attacked, unguibus et rostro, by Mr. Lambert, who declared, that the symptoms of strangulated hernia, were so decided, that no surgeon who knew any thing of his profession, *ought* to mistake them! Mr. L. was then asked by Mr. North to detail those symptoms of strangulation, seeing that they were all so plain and palpable. At this question, Mr. Lambert seemed rather inclined to fight shy, but after a little coaxing on the part of Mr. North, replied that he was not inclined to place so much reliance on the local symptoms as the constitutional. These were obstinate constipation—vomiting—small hard pulse—tenderness of the abdomen. It was observed, however, that these symptoms might one and all exist, totally independent of any herniary tumour, and depend solely upon intestinal inflammation. In opposition to Mr. L's comparative neglect of the local symptoms, it was urged by Mr. North, with great justice, that surgeons in general, are disposed to place very considerable reliance upon them, and Mr. Lawrence, himself, attaches a good deal of importance to the condition of the tumour.

---

12. ICE TO ANEURISMS.

Baron Larrey has lately presented a memoir to the Royal Institute, on the treatment of aortic aneurisms, which consists in the adoption of Valsalva's method —starvation—and the application of ice to the region of the aneurism. Among many instances of success attending this treatment, we may cite the following. A soldier, after a wound, became affected with varicose aneurism of the crural artery. The volume of the tumour exceeded that of a man's fist, and "all operation was impracticable"—foi what reason we are not told. The patient, however, was doomed to death, when the method of Valsalva (starvation, absolute repose, repeated bleedings) and the constant application were tried. Afterwards moxas were applied to the tumour. In the course of a few months, the aneurism was considerably, though very progressively, reduced. It was evident that the parietes of the tumour became gradually thickened. Finally, the canal of the crural artery was completely obliterated, and a cure was effected in the course of a year.

